# The Positive Effect of 6-Gingerol on High-Fat Diet and Streptozotocin-Induced Prediabetic Mice: Potential Pathways and Underlying Mechanisms

**DOI:** 10.3390/nu15040824

**Published:** 2023-02-06

**Authors:** Kunli Wang, Linghua Kong, Xin Wen, Mo Li, Shan Su, Yuanying Ni, Junlian Gu

**Affiliations:** 1 School of Nursing and Rehabilitation, Cheeloo College of Medicine, Shandong University, Jinan 250012, China; 2 College of Food Science and Nutritional Engineering, China Agricultural University, Beijing 100083, China

**Keywords:** 6-gingerol, high-fat diet, prediabetes, gut microbiota, liver

## Abstract

The purposes of the present work are to assess how 6-gingerol (6G) positively influences serum glucose regulation in mice with prediabetes triggered by streptozotocin (STZ) plus a high-fat diet (HFD) and to clarify its underlying mechanisms. An analysis of prediabetic symptoms and biochemical characteristics found that 6G intervention was significantly associated with reduced fasting glucose levels, alleviated insulin resistance, better glucose tolerance, hepatic and pancreatic impairment, and dyslipidemia. For the recognition of the target gut microbiota and the pathways linked to 6G’s hypoglycemic function, a combination of hepatic RNA and 16S rRNA sequencing was employed. Specifically, 6G significantly improved the dysbiosis of the gut microbiota and elevated the relative abundances of *Alistipes*, *Alloprevotella*, and *Ruminococcus_1*. Furthermore, 6G supplementation inhibited gluconeogenesis and stimulated glycolysis by activating the PI3K/AKT axis, which also repressed the oxidative stress through Nrf2/Keap1-axis initiation. In addition, Spearman’s correlation analyses reveal a complex interdependency set among the gut microbiota, metabolic variables, and signaling axes. Taken together, the hypoglycemic effect of 6G is partially mediated by altered gut microbiota, as well as by activated Nrf2/Keap1 and PI3K/AKT axes. Thus, 6G may be used as a candidate dietary supplement for relieving prediabetes.

## 1. Introduction

Recently, with lifestyle changes, the global prevalence of diabetes has reached alarming levels. By 2045, the estimated number of adults suffering from diabetes will be 700 million, and type 2 diabetes mellitus (T2DM) will be the most prevalent form of diabetes [1]. For T2DM, impaired fasting glycemia and impaired glucose tolerance (IGT) constitute the high-risk symptoms of prediabetes [2,3]. Notably, patients with IGT represent over 80% of the entire prediabetic population, and in the absence of appropriate interventions, T2DM progression occurs in more than 70% of IGT cases every year [4,5]. Therefore, effective interventions for prediabetes are a desirable way of delaying or preventing T2DM occurrence. Due to the side effects of antidiabetic drugs, finding and developing natural hypoglycemic substances and adjusting metabolic homeostasis have become safe and feasible nutritional strategies for the prevention of T2DM.

Gut microbiota composition and dysfunction exhibit a tight linkage to the onset and evolution of prediabetes and T2DM [6]. Dysbiosis of gut microbiota has been found to produce peptidoglycan and lipopolysaccharide, which can enter the bloodstream and cause inflammation in the body, ultimately leading to insulin resistance and prediabetes [7]. Interestingly, studies have reported that functional foods and their bioactive constituents (e.g., plant-derived polyphenols) might be capable of altering the composition of gut microbiota, thereby helping regulate host physiology and metabolism [8,9]. Therefore, intervention for gut microbiota using plant active ingredients may be a key tool in prediabetes management. Furthermore, through the mediation of lipid metabolism, glucose homeostasis, and protein synthesis, the PI3K/AKT pathway can play a vital function in cellular physiology, whose imbalance is a cause of T2DM and developing obesity [10]. Nuclear transcription factor erythroid 2-associated factor 2 (Nrf2)/Kelch-like ECH-related protein l (Keap1), the most critical pathway of antioxidant defense, is closely associated with improving the body’s antioxidant capacity and reducing inflammation [11]. This pathway exerts a pivotal effect on T2DM development [12]. Therefore, it is necessary to discover natural active substances that have regulatory effects on both pathways and their downstream related pathways.

As a common spice and herbal medicine, ginger (*Zingiber officinale Roscoe*) has been reported to promote body metabolism, regulate blood glucose, and improve obesity [13,14]. Its chief bioactive constituent is 6G (6-gingerol) [15,16,17], which is capable of entering the blood without causing structural alteration or disruption [18]. Additionally, 6G has been shown to ameliorate HFD-induced dysglycemia in obese rats [19] and to inhibit HFD-induced adipocyte inflammation in obese zebrafish [18]. Thus, 6G is considered a small-molecule compound that can exert metabolic regulatory effects in vivo. However, the mechanism by which 6G improves glycemia in prediabetes is unclear. This study aims to assess 6G’s ameliorating actions on prediabetes induced by HFD/STZ among mice and to clarify the mechanisms behind such actions. The present findings are expected to offer some experimental data on 6G as an ingredient for functional foods.

## 2. Materials and Methods

### 2.1. Preparation of 6G Samples

First, 6G (CAS: 23513-14-6, ≥98% HPLC purity) was procured from Yuanye Biotechnology in Shanghai, China. Following an initial dissolution in 2% dimethyl sulfoxide (DMSO), the 6G was diluted to 2.5 mg/mL using saline and then subjected to a 10 min ultrasonication. This freshly prepared solution was readily usable.

### 2.2. Animals and Experimental Design

The source of the male C57BL/6J mice, which were aged 4 weeks, was the Vital River Laboratory Animal Technology in Beijing, China. We incubated these mice routinely at 25 ± 2 °C with 45–65% RH under a 12 h light and 12 h dark cycle. The mice were all fed and watered ad libitum. Each animal experiment, which was approved by the Ethics Committee of Beijing Vitalstar Biotechnology Co. LTD (Beijing, China; approval code, VST-SY-201912), was conducted following the National Research Council Guidelines.

**Figure 1:**
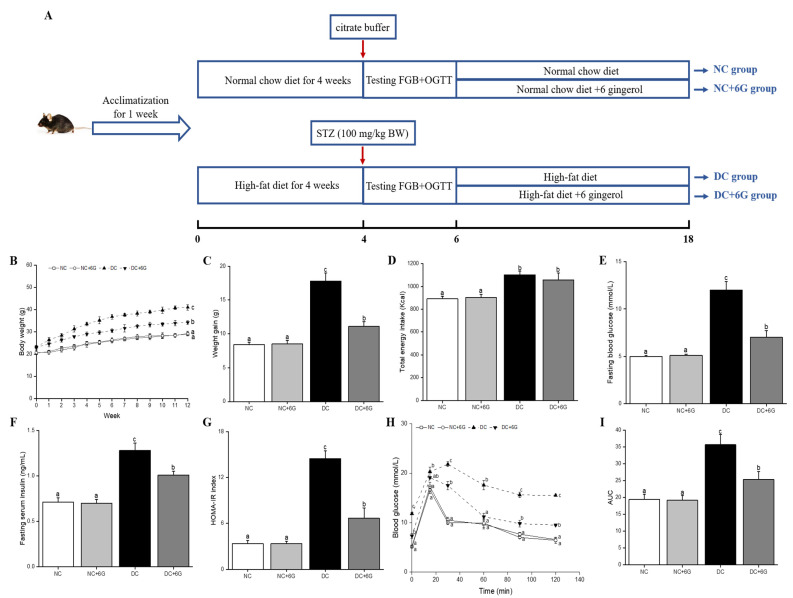
Experimental protocol and design and the effects of 6-gingerol supplementation on body weight, energy intake, and serum glucose metabolism disorder in prediabetic mice. (**A**) Experimental protocol and design, (**B**) body weight, (**C**) weight gain, (**D**) total energy intake, (**E**) fasting blood glucose, (**F**) fasting serum insulin, (**G**) HOMA-IR, (**H**) OGTT curve, (**I**) AUC. Means with different letters on the bar charts indicate significant differences (*p* < 0.05). NC—normal control group; NC+6G—normal mice treated with 6-gingerol; DC—prediabetic control group; DC+6G—prediabetic mice treated with 6-gingerol.

Figure 1A illustrates the holistic design of the experimentation. Following a 1-week acclimatization, the mice were randomly divided into two groups and fed different diets. Supplementary Table S1 details the energy densities and ingredients of the aforementioned diets. For partial mice, after a 6-week feeding of a normal chow diet (NCD; D12450J, with 10 kcal% fat; Research Diets, New Brunswick, NJ, USA), the mice were randomized into two groups. One group continued to be fed NCD (NC group, *n* = 8), and the other group was fed NCD supplemented with 6-gingerol (NC+6G group, *n* = 8). For the remaining mice, a 4-week feeding of HFD (D12492, supplemented with 60 kcal% fat, Research Diets, New Brunswick, NJ, USA) was implemented initially, and after a 12 h fast, the prediabetic mice were induced by administering the STZ solution (freshly prepared; 100 mg/kg body mass) inside the peritoneum. Fasting blood glucose (FBG) was performed on the mice on day 14, post-injection. Following the sampling of venous blood from the murine tail, a blood glucose meter (Ascensia, Shanghai, China) was utilized to determine the levels of blood glucose. In the current work, mice with FBG in the 3.2–6.2 mM range and 2-h postprandial glucose (2h-PG) in the 7.8–11.1 mM range were deemed as fulfilling the prediabetes (IGT) criteria with two tests and a screening [20,21]. The mice satisfying the prediabetes criteria were randomized into the DC (prediabetic control) group (*n* = 8) receiving HFD and 2 other prediabetic groups receiving HFD plus an additional 10 mg/kg·body weight (BW) 6-gingerol (*w*/*w*, HFD+6G, *n* = 8). During the dosage choice, our prior work was consulted [22]. Throughout the experiment, food ingestion was documented twice weekly, while body mass was documented on a weekly basis. One week before the experimental completion, the mice were gavaged. Six hours later, they were arranged in the metabolic cages, and, following defecation, fecal samples were directly gathered into disinfected conical tubes. Afterward, liquid nitrogen was used to immerse these fecal samples, followed by a −80 °C preservation for subsequent analyses. Upon experimental completion, blood was sampled from the retro-orbital vascular plexus following an overnight abrosia, which was subjected to a 10 min dissociation (3000 rpm) at 4 °C to derive the sera. Instantly thereafter, the sera were preserved at −80 °C for more detailed biochemical assays. After blood collection, the mice were sacrificed, and their tissues and organs were harvested.

### 2.3. Biochemical Analysis

An automatic biochemistry analyzer (Hitachi, Tokyo, Japan) was utilized to determine the levels of serum biochemical variables, such as the total cholesterol (TC), triglyceride (TG), high- and low-density lipoproteins (HDL/LDL), aspartate aminotransferase (AST), alanine aminotransferase (ALT), and FBG. Commercially available ELISA kits (Jiancheng Bioengineering Institute, Nanjing, China) were utilized to examine the levels of fasting serum insulin, IL-6 (interleukin 6), and TNF-α (tumor necrosis factor α). Assessment of serum LPS concentrations was accomplished following the instructions of a commercially available ELISA kit (Enzyme-linked Biotechnology, Shanghai, China).

### 2.4. Oral Glucose Tolerance Test (OGTT)

Three days prior to experimental completion, an OGTT was conducted according to a prior procedure [23]. Briefly, a 25% solution of glucose dextrose (2 g/kg·BW) was orally administered to the mice fasted for 6 h. Separately at 0, 15, 30, 60, 90, and 120 min after glucose administration, the levels of blood glucose were assessed using a blood glucose meter. For the glucose tolerance evaluation in this study, the areas under the curve (AUCs) of the blood glucose levels were estimated over a 120 min period.

### 2.5. Histology and Immunofluorescence Staining for Tissue Section Analysis

Following the killing of the mice, their hepatic and pancreatic tissues were immobilized in paraformaldehyde (4%), paraffin-embedded, and subsequently sectioned to a 5 µm thickness, followed by hematoxylin and eosin (H&E) staining as per the standard procedure. Thereafter, an Eclipse-ci microscope (Nikon, Tokyo, Japan) was utilized to observe the slides and acquire micrographs. For immunofluorescence, the paraffin-embedded pancreatic sections were deparaffinized, antigen recovered, heated, and blocked. After overnight incubation of the slides using Mouse Anti-Insulin (primary) antibody at 4 °C, an extra 2 h incubation proceeded with Anti-Mouse IgG (secondary; Alexa Fluor 488) antibody at ambient temperature. The samples were coated with DAPI (ProLong^®^GoldAntifade Reagent, Waltham, Massachusetts, USA) anti-fading reagent, and then a BK-FL fluorescent microscope (Chongqing, China) was utilized for their observation.

### 2.6. Real-Time PCR (RT-PCR) Analysis

Extraction of the total RNA from the hepatic tissue was accomplished following the protocol of TRIZOL reagent (Invitrogen, Carlsbad, CA, USA). Quantitative and qualitative ratiometric analyses of the RNA were conducted with the aid of a Nanodrop 2000 (Thermo Scientific, Wilmington, NC, USA). Reverse transcription of the RNA into cDNA was then carried out using a corresponding High-Capacity kit from Tiangen Biotech. SYBR Green (Shanghai, China) was used to conduct RT-PCR, with which the expression of DEG was relatively quantified. PCR conditions were as follows: 10 min at 95 °C, 10 s at 95 °C, 10 s of annealing at 60 °C, and 10 s of extension at 72 °C, a total of 40 cycles [24]. Regarding the relative mRNA levels of the genes, the 2^−ΔΔCT^ approach was adopted for their estimation, while β-actin was employed as an internal reference for their normalization. Table S2 details the relevant primers.

### 2.7. Western Blot Analysis

The experiments were conducted according to a previously reported study [25]. A 100 mg:1 mL lysis buffer involving a protease suppressor was used for the dissolution of the hepatic tissue. The determination of the protein levels was accomplished per the instructions of a BCA protein assay kit (Pierce, Rockford, AZ, USA). This was followed by isolation of the lysates on the SDS-PAGE gels and subsequent shifting onto the PVDF membranes (0.22 µm). A 1 h blockage of these membranes proceeded in Tris-buffered saline (TBS) involving skimmed milk (5%) and Tween-20 (1%) at room temperature, followed by a 1 h incubation with monoclonal primary antibodies against AKT and p-AKT (1:1000; Beyotime Biotechnology, Shanghai, China) as well as against β-actin, G6P, GK, Nrf2, PEPCK, and Keap1 (Cell Signaling Technology, Danvers, Massachusetts, USA). After incubation, the membranes were thrice washed, followed by an extra 2 h incubation using a 1:2000 dilution of anti-rabbit secondary antibody (Cell Signaling Technology) at 37 °C. Visualization and assessment of Western blot images were accomplished using densitometric scanning (Image Quant TL7.0, GE Healthcare, Chicago, Illinois, USA), where the loading control adopted was β-actin.

### 2.8. Gut Microbiota Analysis

A Mag-Bind Soil DNA Kit (E.Z.N.A; OMEGA, GA, USA) was utilized to extract the genomic DNA from the fecal specimens. Thereafter, to exploit the primers 806R (50-GGACTACHVGGGTWTCTAAT-30) plus 338F (50-ACTCCTACGGGAGGCAGCAG-30), PCR amplification was conducted targeting the 16S rRNA gene’s V3–V4 regions. The PCR products were subjected to purification via a QIAquick PCR Purification Kit (QIAGEN, Valencia, USA). With a MiSeq platform (Illumina, San Diego, CA, USA), sequencing and assessment of the purified amplicons were carried out.

QIIME (V 1.91) was used as a quality filter for the raw FASTQ files after the accomplishment of sequencing. Then, the operational units (OTUs) were clustered by employing UPARSE (7.0.1090, http://www.drive5.com/uparse/, accessed on 10 March 2022), where the similarity cutoff was set at 97%. Mothur V.1.30.1 was utilized to conduct the alpha-diversity analysis. Chao1 and Ace were adopted to estimate the richness of the communities, while the Simplon and Shannon indices were adopted for the diversity evaluation. The differences between microbial communities were quantified using principal coordinate analysis (PCoA). For the effect-size assessments of the relevant abundance of specific differential bacteria, linear discriminant analysis (LDA) scores were derived on the basis of the linear discriminant analysis effect size (LEfSe); http://huttenhower.sph.harvard.edu/galaxy/root?tool_id=lefse_upload, accessed on 11 March 2022) was used (*p* < 0.05 and LDA score > 3.0) [26]. Data were analyzed online on the freely available Majorbio Cloud Platform (www.majorbio.com, accessed on 10 March 2022).

### 2.9. Statistical Analysis

Data were all expressed as means ± SDs (standard deviations). Univariate analysis of variance (ANOVA) and Tukey’s post hoc test were employed for the analysis of all the experimental outcomes. Furthermore, differences at *p* < 0.05 indicated statistical significance. Statistical analysis was performed using SPSS 20.0 (IBM, New York, NY, USA).

## 3. Results

### 3.1. Effects of 6-Gingerol Supplementation on Body Weight and Energy Intake

Figure 1B shows the body-mass changes for every group following the prediabetic mice modeling. We found persistent weight gain among the DC group mice compared with the NC and NC+6G groups, and 6G supplementation significantly prevented weight gain (Figure 1C). In addition, 6G supplementation had no effect on the total energy intake in the normal and prediabetic mice during the 12-week experimental period (Figure 1D).

### 3.2. Effects of 6-Gingerol Supplementation on Blood Glucose Metabolism

For the hypoglycemic efficacy assessment of the 12-week treatment with 6G, we determined the FBG, OGTT, and fasting serum insulin (FSI) of every group. The DC group exhibited pronouncedly higher levels of FBG compared to the NC group (12.06 ± 0.92 vs. 5.01 ± 0.12 mmol/L). Mice were deemed as suffering from T2DM when their FBG exceeded 11.1 mmol/L [27]. Therefore, our findings agree with the former work [28] that long-term continuous feeding of HFD following administration of streptozotocin in the DC group can lead to the development from prediabetes to T2DM. The DC+6G mice exhibited drastically decreased FBG than the DC mice. Additionally, higher HOMA-IR and FSI were noted among the DC mice compared to the NC mice, while supplementation with 6G led both of the metrics to decline markedly (Figure 1F,G). Furthermore, the results of the OGTT and AUC demonstrate a serious impairment of glucose tolerance among the DC mice (Figure 1H,I). Supplementation with 6G significantly improved glucose tolerance and insulin resistance.

### 3.3. Effect of 6-Gingerol Supplementation on Serum Parameters

The serum ALT, AST, LDL, TG, and TC levels in the DC group were pronouncedly higher than those in the NC group (Table 1), while 6G treatment markedly reduced the levels of the other indicators except LDL. The level of HDL had also been improved by 6G treatment. Furthermore, 6G led to significantly decreased IL-6, LPS, and TNF-α levels in both the normal and prediabetic mice. This suggests that 6G not only improves the health status of normal mice but also alleviates HFD/STZ-induced metabolic endotoxemia and systemic low-grade inflammation.

### 3.4. Effect of 6-Gingerol Supplementation on the Histopathology of Liver and Pancreatic Tissues

**Figure 2:**
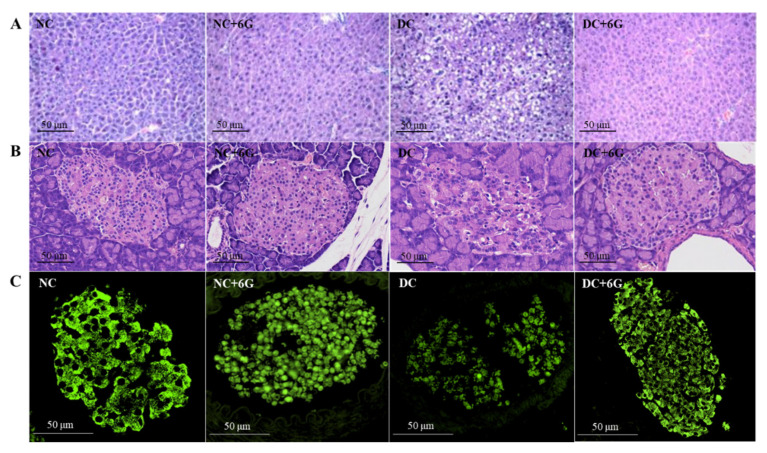
Effects of 6-gingerol supplementation on histopathology of liver and pancreatic tissues in prediabetic mice. (**A**) Liver (H&E stain 200× magnification), (**B**) pancreas (H&E stain 200× magnification), (**C**) immunochemical analysis of pancreas beta cells. Pancreatic sections were stained with insulin (green, 400× magnification). NC—normal control group; NC+6G—normal mice treated with 6-gingerol; DC—prediabetic control group; DC+6G—prediabetic mice treated with 6-gingerol.

As shown in Figure 2A, the NC mice had normal, neat, and clear hepatic histology, and there were obvious nuclei in the center of the hepatocytes. In addition, 6G supplementation in normal mice did not adversely affect their liver tissue. Serious impairments of liver tissue were noted among the DC mice, including massive hepatocyte steatosis, inflammation infiltrates, and cytoplasmic vacuolization. However, 6G supplementation greatly attenuated the histopathological changes (Figure 2A). These results suggest that 6G supplementation can attenuate hepatocyte injury induced by a high-fat diet and STZ to a certain extent.

According to the results of the pancreatic H&E staining, the pancreatic islet structure was normal for the NC+6G and NC groups (Figure 2B). The DC group had an atrophic structure of the islets, necrotic β-cells in the pancreas, and a destroyed β-cell population. However, the islet structure impairment and necrosis of the β-cells were alleviated prominently with 6G supplementation. Immunofluorescence staining of the pancreatic tissue showed that the pancreatic islets in the NC group and NC+6G group had round or ovoid cell clusters scattered among healthy glandular follicle cells with clear cell borders (Figure 2C). The DC group exhibited a distinctly reduced area and brightness of the pancreatic islets in contrast to the NC group, and the pancreatic islet morphology was irregularly atrophied, suggesting that the pancreatic islet β-cells were apoptotic. The DC+6G group displayed increases in the islet brightness and area in contrast to the DC group, suggesting that the intervention of 6G had a protective effect on the pancreatic islet β-cells.

### 3.5. Effects of 6-Gingerol Supplementation on Hepatic-Glucose-Metabolism-Related and Oxidative-Stress-Pathway-Related mRNA and Protein Expression

**Figure 3:**
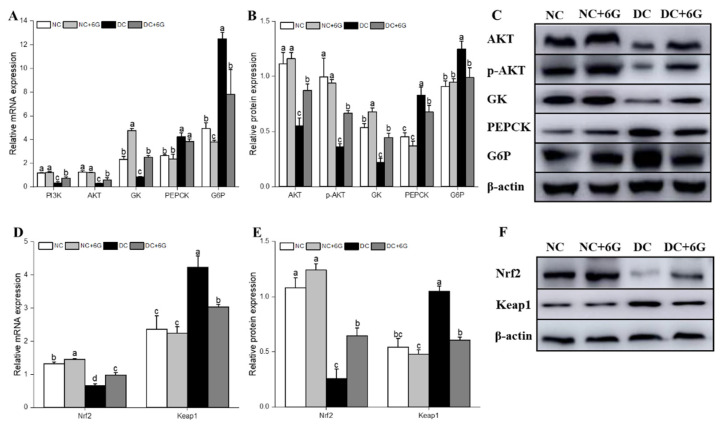
6-gingerol regulated the hepatic-glucose-metabolism-related and oxidative-stress-pathway-related mRNA and protein expression in prediabetic mice. (**A**) Hepatic-glucose-metabolism-related mRNA expression, (**B**,**C**) hepatic-glucose-metabolism-related protein expression, (**D**) oxidative-stress-pathway-related mRNA expression, (**E**,**F**) oxidative-stress-pathway-related protein expression. Means with different letters on the bar charts indicate significant differences (*p* < 0.05). NC—normal control group; NC+6G—normal mice treated with 6-gingerol; DC—prediabetic control group; DC+6G—prediabetic mice treated with 6-gingerol.

It is well established that the PI3K/AKT axis is implicated in insulin-mediated hepatic glucose metabolism, while GK, PEPCK, and G6P are key proteins in glycolysis and gluconeogenesis. The DC+6G group exhibited pronouncedly upregulated PI3K and AKT mRNA levels in contrast to the DC group after the 6G treatment (Figure 3A). At the protein level (Figure 3B,C), the expression of both AKT and p-AKT after 6G treatment was significantly upregulated. GK, the key protein for glycolysis, was also upregulated after 12 weeks of 6G intervention, while key proteins for gluconeogenesis PEPCK and G6P were downregulated after 6G supplementation. Notably, the protein expression level of GK was elevated in the normal mice supplemented with 6G.

Nrf2/Keap1 plays a protective role in antioxidant damage mainly by upregulating antioxidant genes and reducing redox stress. Activation of Nrf2 may be a way to ameliorate oxidative stress. After 12 weeks of 6G intervention, both the protein and mRNA expression of Nrf2 and Keap1 were improved in the prediabetic mice (Figure 3D–F). In addition, 6G supplementation also improved the hepatic protein and mRNA levels of Nrf2 and Keap1 among the normal mice.

### 3.6. 6-Gingerol Supplementation Changed the Gut Microbiota

**Figure 4:**
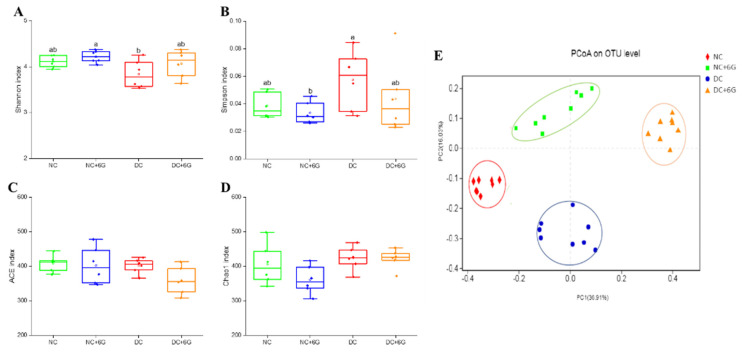
Changes in the structure and composition of gut microbiota: (**A**–**D**) the diversity (Shannon and Simpson) and the richness (ACE and Chao1) of the gut microbiota among different groups; (**E**) principal co-ordinates analysis (PCoA) on the OTU level. Means with different letters on the bar charts indicate significant differences (*p* < 0.05). NC—normal control group; NC+6G—normal mice treated with 6-gingerol; DC—prediabetic control group; DC+6G—prediabetic mice treated with 6-gingerol.

To investigate the underlying mechanisms of the hypoglycemic effect of 6G, this study assesses the effects of 6G supplementation on the composition and relative abundance of gut microbiota with 16S rRNA sequencing. A total of 1313 859 sequences were generated by setting the mean length to 401–440 base pairs (Figure S1A). A total of 730 OTUs were derived according to 97% similarity (Figure S1B), which can be categorized as 140 genera, 56 families, 32 orders, 19 classes, and 10 phyla. As suggested by the Shannon and rarefaction graphs of the samples (Figure S1C,D), the bacterial communities were detected clearly and distributed homogeneously, and the amount of data sequenced was sufficient. Supplementation with 6G can significantly change gut microbiota diversity (both Shannon and Simpson) (Figure 4A,B). However, insignificant intergroup differences were found in the ACE and Chao1 indices of the gut microbiota (Figure 4C,D), suggesting that 6G had no effect on richness. Afterward, a principal co-ordinates analysis (PCoA) was accomplished on the unweighted UniFrac distances (Figure 4E). As revealed by the results, the gut microbiota composition was significantly altered under the combined HFD/STZ induction and 6G supplementation.

**Figure 5:**
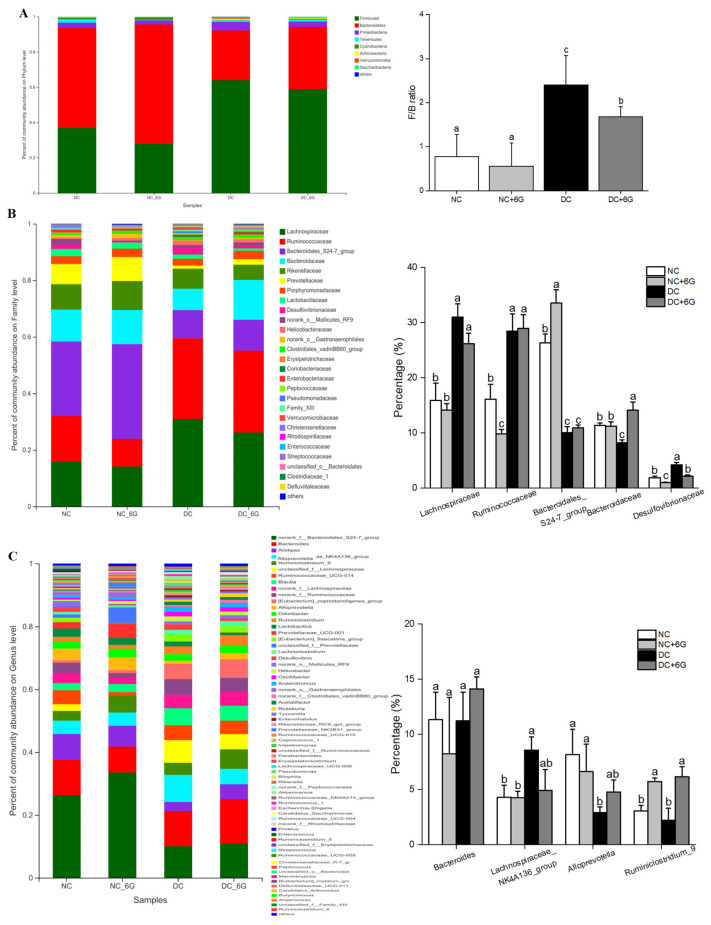
The relative abundance of gut microbiota at the phylum, family, and genus levels. Percentage of community abundance in NC, NC+6G, DC, and DC+6G groups at the level of (**A**) phylum, (**B**) family, and (**C**) genus. NC—normal control group; NC+6G—normal mice treated with 6-gingerol; DC—prediabetic control group; DC+6G—prediabetic mice treated with 6-gingerol. The diverse letters represent a significant difference (*p* < 0.05). F/B—Firmicutes: Bacteroidetes.

Further, the gut microbiota structures in the four groups were analyzed at different classification levels (Figure 5). Combined HFD/STZ induction led to significant phylum-level alteration of the dominant microbial communities (*Bacteroidetes and Firmicutes*) (Figure 5A), while after supplementing 6G, the abundance of the dominant microflora returned to normal. Additionally, the *Firmicutes*-to-*Bacteroidetes* (F/B) ratio was downregulated after 6G intervention in the normal or model mice (Figure 5A). At the family level, higher abundances were noted in *Lachnospiraceae*, *Ruminococcaceae*, and *Desulfovibrionaceae* following treatment with HFD/STZ (Figure 5B), while the abundances of *Bacteroidales_S24-7_group* and *Bacteroidaceae* were lower. The abundances of *Bacteroidaceae* and *Desulfovibrionaceae* were significantly up- and downregulated, respectively, after 6G intervention. According to the genus-level observations, the NC+6G and DC+6G groups exhibited considerably higher relative abundances of *Ruminiclostridium_9* when compared to the NC and DC groups (Figure 5C).

### 3.7. The Changes in Key Phylotypes of the Gut Microbiota That Responded to 6-Gingerol Supplementation

**Figure 6:**
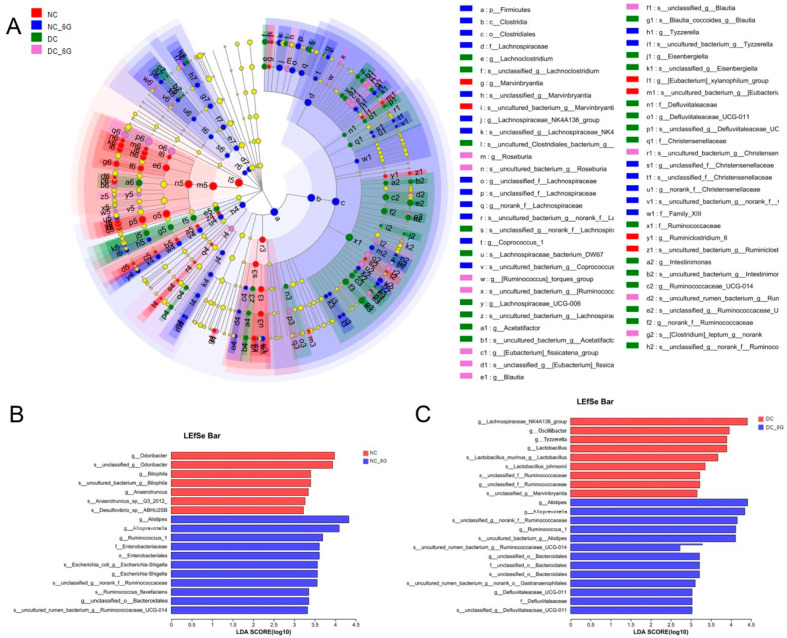
Key phylotypes of the gut microbiota that responded to 6-gingerol supplementation in the mice. (**A**) Cladogram generated from LEfSe analysis showing the relationship between taxa (the levels represent, from the inner to outer rings, phylum, class, order, family, and genus); (**B**) linear discriminant analysis (LDA) score for taxa differing between NC and NC+6G; (**C**) linear discriminant analysis (LDA) score for taxa differing between DC and DC+6G. An LDA score greater than 3 indicates a higher relative abundance in the corresponding group than in the other group.

The cladogram generated from the LEfSe analysis indicated that 6-gingerol supplementation altered the bacterial taxa specifically (Figure 6A). In addition, LDA scoring was performed to identify discriminative features (Figure 6B,C). Among the NC mice, we observed abundance elevations in a few bacterial genera, such as the genera *Bilophila*, *Odoribacter*, and *Anaerotruncus*. As for the DC group, enrichment of the gut microbiota was noted in the genera *Oscillibacter* and *Tyzzerella*. Furthermore, both the NC+6G and DC+6G groups are characterized by a higher content of the *Alistipes*, *Alloprevotella*, and *Ruminococcus_1* genera.

### 3.8. Correlations among the Critical Gut Microbiota, Biochemical Parameters, and Signaling Pathways

**Figure 7:**
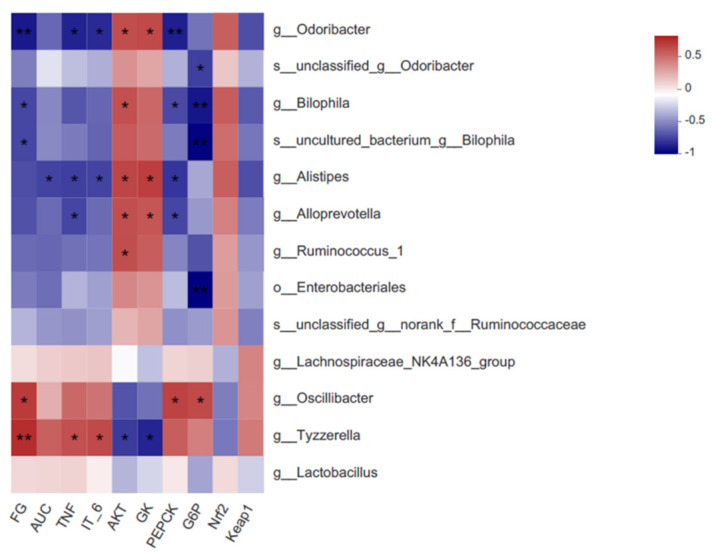
Correlation between biochemical parameters, liver gene expression, and key communities of gut microbiota obtained from LEfSe results. Spearman’s correlation coefficients and *p* values for the correlations are calculated (* *p* < 0.05, ** *p* ≤ 0.01). A positive correlation is shown in red, and a negative correlation is shown in blue.

To probe deeper into the correlations among the crucial gut microbiota, biochemical parameters, and key hepatic gene expression, a Spearman correlation assessment was performed (Figure 7). As revealed by the assessment, there were negative associations of *Alistipes*, *Alloprevotella*, and *Odoribacter* with TNF-α, and *Alistipes* also showed a negative correlation with AUC and IL-6. In addition, *Odoribacter*, *Bilophila*, and *uncultured_bacterium_g_Bilophila* showed a negative correlation with FG, and *Oscillibacter* and *Tyzzerella* showed a positive correlation with FG.

As for the correlation of gut microbiota with hepatic gene expression, we noticed significant positive associations of *Alloprevotella, Alistipes*, and *Ruminococcus_1*, which were enriched with 6G supplementation, with the level of AKT. *Alloprevotella* and *Alistipes* also showed a negative correlation with PEPCK expression. Moreover, *Odoribacter* was linked significantly positively to AKT and GK expression and showed a negative correlation with PEPCK expression.

## 4. Discussion

During the development of diabetes, prediabetes is a high-risk state, and with appropriate interventions, the progression to diabetes can be reversed, and blood glucose can even be restored to normal levels [29]. Numerous studies have demonstrated the metabolic modulating function of ginger [13,14,24,30]. Our previous study showed that ginger oleoresin has the function of regulating blood glucose metabolism in mice, and 6G comprises the biggest proportion of active ingredients in ginger oleoresin [22]. As far as we know, the present work is the first to offer evidence that supplementation with 6G, an active substance in ginger, helps to improve HFD/STZ-induced prediabetes.

In this study, progressive weight gain was noted among the DC mice following HFD feeding, while supplementation with 6G was effective in controlling weight gain, independent of food intake. Agreeing with the former findings of [31], 6G’s hypoglycemic activity is proven by the evident declines in AUC and FG. In addition, 6G was effective in improving liver and pancreas damage. In mice with prediabetes triggered by HFD/STZ, 6G supplementation significantly restored glucose tolerance, ultimately avoiding the worsening of prediabetes. Next, this study has further investigated the underlying mechanism by which 6G improves blood glucose levels in prediabetic mice by revealing the effects of 6G on the expression of hepatic key genes and the composition of gut microbiota.

The liver is a crucial organ for keeping bodily glucose homeostasis [32]. Glucose metabolism in the liver consists of glycolysis, gluconeogenesis, glucose transport, glycogen synthesis, and catabolism [33]. Through the translocation facilitation of glucose transporter 4, glucose transport can be upregulated by 6G [34]. However, evidence for the regulation of other key glucose metabolic pathways by 6G is still lacking. Regulation of these key pathways is achieved via the insulin-mediated PI3K/AKT axis [35]. Hence, we have further unraveled the possible mechanisms for glucose metabolism regulation by 6G by exploring how supplementation with 6G specifically influences the PI3K/AKT axis as well as its downstream effectors.

As indicated by extensive evidence, the foremost factors in the PI3K/AKT axis are AKT and PI3K [35]. For instance, AKT has high levels of expression in the liver and other conventional insulin target tissues, and its positive role in reducing blood glucose and improving insulin sensitivity has been reported [36]. Both of these factors were upregulated in our current study, suggesting PI3K/AKT-axis initiation by the supplemental 6G. Then, the levels of the critical genes in glycolysis (e.g., G6P) and gluconeogenesis (e.g., GK and PEPCK) were determined to further elucidate the biological processes and target genes implicated in 6G’s hypoglycemic function. Studies have shown that through expression regulation of these enzymes, dietary intervention can enhance glucose metabolism [37,38]. For example, by regulating the hepatic levels of glucose-metabolizing enzymes (e.g., PEPCK and G6P), undaria pinnatifida polysaccharides reduced blood glucose [39]. Through the regulation of hepatic G6P and GK levels, millet dietary intervention regulated blood glucose [40]. In our work, upregulation of GK was noted in the hepatic tissues for the 6G intervention group, implying the proglycolytic function of 6G supplementation in the diabetic murine liver tissues, which was achieved with glycolysis upregulation. Meanwhile, G6P and PEPCK expression was downregulated in the liver, indicating that 6G also downregulated hepatic gluconeogenesis.

Given that oxidative stress exacerbates T2DM, antioxidant therapy has gained more attention [41]. It has been shown that the Nrf2 pathway is inhibited in response to oxidative stress stimuli and impaired islet function is exacerbated, possibly mediated by PI3K/AKT [42]. In addition, natural active components in plants, such as sulforaphane, can effectively modulate Nrf2 [43]. In this study, Nrf2 and Keap1, key genes in the Nrf2 pathway, were regulated after 6G intervention, indicating that the Nrf2/Keap1 pathway was improved.

There is now accumulating evidence that the onset and progression of T2DM are closely linked to the gut microbiota [44,45], whose role in prediabetic disease development is thus pivotal. Therefore, apart from the possible direct effect of 6G on the liver in regulating hepatic glucose metabolism, the effect of 6G on gut microbiota was also investigated in the case of prediabetic mice. Here, 6G was found to restore the HFD/STZ-disrupted composition of the gut microbiota and facilitate certain specific bacterial growth in the mice intaking HFD and NCD. The abundance of *Bacteroidaceae* in the prediabetic model mice was upregulated after 6G intervention. Downregulation of *Bacteroidaceae* may contribute to the occurrence of T2DM [46]. In addition, 6G supplementation restored the HFD/STZ-induced enrichment of *Desulfovibrionaceae*, which cause intestinal inflammation through the production of endotoxins (such as LPS). This finding agrees with previous research reporting the ability of a plant-based diet to decrease *Desulfovibrionaceae* abundance upregulation due to HFD [14,47]. Furthermore, 6G intervention altered key phylotypes in each group at the genus level. As mentioned in the Results section, three species, the *Alistipes*, *Alloprevotella*, and *Ruminococcus_1* genera, were present in the key communities in the NC+6G and DC+6G groups, suggesting that 6-gingerol supplementation significantly upregulated three OTUs. Sshort-chain fatty acid (SCFA)-producing bacteria like *Alistipes*, *Alloprevotella*, and *Ruminococcus_1* [10,48,49,50] were enriched after 6G supplementation. Inverse correlations of these SCFA-producing bacteria with insulin resistance, inflammation, and obesity have been reported [51,52]; moreover, these bacteria may enhance host antidiabetic effects.

As demonstrated by the results of a correlation analysis of environmental factors, the specific gut microbiota were negatively associated with the physiological and biochemical correlates of prediabetes and were associated with factors pertaining to PI3K/AKT expression, gluconeogenesis, and glycolytic pathways. Therefore, our outcomes imply that apart from impacting prediabetes markers, gut microbiota may also be correlated with critical hepatic gene expressions. As supported by increasing evidence, gut microbiota (e.g., *Alistipes*, *Rikenella*, and *Odoribacter*) influence key hepatic gene expressions and are positively linked to factors pertaining to the PI3K/AKT and AMPK/SIRT1 axes [10,53,54]. In the present study, the upregulation of *Alistipes* and *Alloprevotella* and the downregulation of *Odoribacter* all alleviated prediabetic symptoms, upregulated the PI3K/AKT and glycolytic pathways, and downregulated glycoisomeric pathways. However, no correlation between the Nrf2/Keap1 pathway and gut microbiota was found. It may be that the 6G intervention affected the metabolism and composition of the gut microbiota and that there was portal venous transport of the metabolites to the liver, which has an impact on the expression of key genes. However, the metabolites of 6G after metabolism by the gut microbiota and the role of metabolites in the regulation of blood glucose still need to be further explored.

The last thing to point out is that although 6G has a good ability to regulate blood glucose, the current experimental results do not prove that 6G can be used in clinical trials immediately. The basic characteristics of 6G pharmacokinetics such as absorption and metabolism in vivo need to be further explored. On the basis of these results, we will explore the possibility of 6G being developed into drugs or functional foods.

## 5. Conclusions

Conclusively, we discovered herein that 6G intervention reduced HFD/STZ-induced weight gain and improved metabolic syndrome symptoms such as hyperglycemia, lowered glucose tolerance, dyslipidemia, insulin resistance, damage of liver and pancreatic tissues, and low-grade systemic inflammation. The 16S rRNA sequencing analysis demonstrated that 6G supplementation led to prominent alterations in the gut microbiota composition, including increases in *Bacteroidaceae*, *Ruminiclostridium_9*, *Alistipes*, *Alloprevotella*, and *Ruminococcus_1* and decreases in *Desulfovibrionaceae*. In addition, 6G regulated blood glucose through PI3K/AKT pathway initiation and suppression of the relative PEPCK and G6P levels, while having a facilitative effect on the Nrf2/Keap1-mediated antioxidant pathway. This evidence suggests that 6G does not exert its protective effect against prediabetes through a single pathway but rather through the body’s overall metabolic balance through the regulation of gut microbiota and the critical hepatic gene levels. Therefore, 6G has great potential as an adjuvant therapy strategy for prediabetes. In addition, using the present study as a basis, further research is needed to explore the metabolites of 6G after metabolism by gut microbiota and the role of metabolites in the regulation of blood glucose.

## Figures and Tables

**Table 1:** Serum biochemical analyses from NC and DC mice with or without 6G treatment.

Group	NC	NC+6G	DC	DC+6G
TC (mmol/L)	3.41 ± 0.12 ^a^	3.27 ± 0.08 ^a^	4.57 ± 0.18 ^c^	3.91 ± 0.18 ^b^
TG (mmol/L)	0.33 ± 0.05 ^b^	0.14 ± 0.02 ^a^	0.68 ± 0.06 ^c^	0.27 ± 0.02 ^a^
HDL (mmol/L)	2.72 ± 0.10 ^ab^	3.51 ± 0.13 ^c^	2.59 ± 0.12 ^a^	2.93 ± 0.13 ^b^
LDL (mmol/L)	0.45 ± 0.07 ^a^	0.58 ± 0.06 ^a^	0.91 ± 0.05 ^b^	1.00 ± 0.10 ^b^
ALT (U/L)	32.80 ± 5.02 ^bc^	18.78 ± 1.23 ^a^	40.25 ± 2.28 ^c^	30.25 ± 2.17 ^b^
AST (U/L)	119.84 ± 6.40 ^ab^	114.75 ± 5.47 ^ab^	131.52 ± 8.08 ^c^	105.51 ± 2.24 ^a^
LPS (EU/L)	17.01 ± 0.22 ^a^	15.80 ± 0.86 ^a^	23.60 ± 1.05 ^c^	20.12 ± 0.30 ^b^
TNF-α (ng/mL)	64.59 ± 8.71 ^a^	51.33 ± 3.62 ^a^	125.28 ± 10.74 ^c^	83.19 ± 6.45 ^b^
IT-6 (ng/mL)	77.56 ± 5.22 ^a^	71.03 ± 4.97 ^a^	146.17 ± 9.84 ^c^	116.16 ± 3.83 ^b^

Data are presented as mean ± SD. Diverse letters in the same row represent significant difference, *p* < 0.05. NC—normal control group; NC+6G—normal mice treated with 6-gingerol; DC—prediabetic control group; DC+6G—prediabetic mice treated with 6-gingerol.

## Data Availability

All data that support the findings of this study are available from the corresponding author on reasonable request.

## Supplementary Materials

The following supporting information can be downloaded at https://www.mdpi.com/article/10.3390/nu15040824/s1. Figure S1: DNA raw data and comparison of the OTUs of intestinal bacteria of mice (A) DNA sequencing number and length; (B) a Venn diagram was generated to describe the common and unique OTUs among the four groups; (C) the rarefaction curves; (D) the Shannon curves of the samples. NC—normal control group; NC+6G—normal mice treated with 6-gingerol; DC—prediabetic control group; DC+6G—prediabetic mice treated with 6-gingerol. Table S1: Ingredients and energy densities of experimental diets. Table S2: The primary sequence used for quantitative real-time polymerase chain reaction.
