# Supplementary material for: The Positive Effect of 6-Gingerol on High-Fat Diet and Streptozotocin-Induced Prediabetic Mice: Potential Pathways and Underlying Mechanisms
Source: Nutrients. 2023 Feb 6;15(4):824. doi: 10.3390/nu15040824 (PMC9968036; doi:10.3390/nu15040824)
Supplement: Supplementary file 1 [file nutrients-15-00824-s001.zip › nutrients-2177237-supplementary.pdf]

Table S1. Ingredients and energy densities of experimental diets.

Ingredients	D12450J		D12492	
	g%	kcal%	g%	kcal%
Casein	0.190	0.198	0.258	0.198
L-cystine	0.003	0.003	0.004	0.003
Sucrose	0.336	0.351	0.094	0.072
Corn starch	0.299	0.312	-	-
Lodex 10 sugar	0.033	0.035	0.162	0.124
Cellulose	0.047	0.000	0.065	0.000
Soybean oil	0.024	0.056	0.032	0.056
Lard	0.019	0.045	0.317	0.547
Mineral mixture	0.047	0.000	0.065	0.000
Hydrocholine tartrate	0.002	0.000	0.003	0.000
Vitamin mixture	0.001	0.000	0.001	0.000
Dye	0.000	0.000	0.000	0.000
Energy（kcal/g diet）	3.82		5.21	

Table S2. The primary sequence used for quantitative real-time polymerase chain reaction.

Gene	Forward	Reverse
PI3K (p110)	GGAGGCTGAATCTCTTGACCTG	TTACCCACCGTGCTCTGCTG
AKT	CGTGTGGCAAGATGTGTATGAG	GTGATGGTGATCATCTGAGCTGT
GK	TGGCCTAATGAAAGCTGGGG	AGCACCCTGTTCCATACGTG
PEPCK	GGATGTGGCCAGGATCGAAA	ATACATGGTGCGGCCTTTCA
G6P	AATGAACGTGCTCCACGACT	CTGCCACCCAGAGGAGATTG
Keap	CTTCGGGGAGGAGGAGTTCT	GGGCAGTCGTATTTGACCCA
Nrf2	CCATTTGGGCTAGTTTCTGTGTA	AGTTTGCTTTTAGCCAGATGTCA
β-actin	GGTCAGAAGGACTCCTAT	TGTCGTCCCAGTTGGTAACA

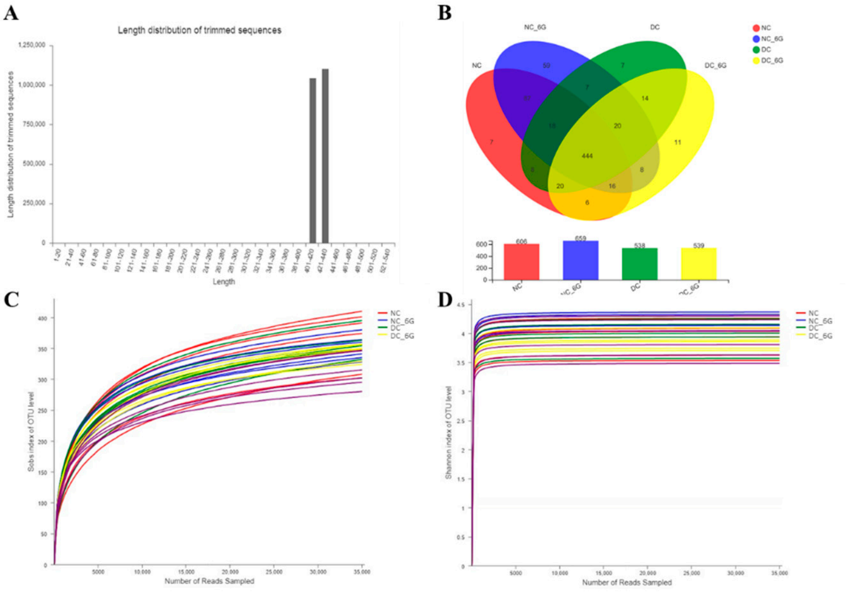

Figure S1. DNA raw data and comparison of the OTUs of intestinal bacteria of mice (A) DNA sequencing number and length; (B) A Venn diagram was generated to describe the common and unique OTUs among the 4 groups; (C)The rarefaction curves; (D) The shannon curves of samples. NC, normal control group; NC+6G, normal mice treated with 6-gingerol; DC, prediabetic control group; DC+6G, prediabetic mice treated with 6-gingerol.
